# Effects of *Allium mongolicum* Essential Oil on Meat Quality and Flavor-Related Free Amino Acids in Finishing Lambs

**DOI:** 10.3390/ani16101559

**Published:** 2026-05-21

**Authors:** Khas Erdene, Xiaoyuan Wang, Yaxing Zhao, Qina Cao, Yankai Zheng, Changjin Ao, Chen Bai

**Affiliations:** 1 Key Laboratory of Animal Feed and Nutrition of Inner Mongolia Autonomous Region, College of Animal Science, Inner Mongolia Agricultural University, Hohhot 010018, China; 2 Inner Mongolia Academy of Agricultural & Animal Husbandry, Hohhot 010031, China

**Keywords:** carcass traits, drip loss, glutamic acid, *longissimus thoracis*, phytoadditives, volatile compounds

## Abstract

The objective of this study was to evaluate the effects of dietary *Allium mongolicum* Regel essential oil (AMO) supplementation on growth performance, carcass traits, meat quality, and muscle amino acid profile in finishing lambs. A total of twenty male Dorper × Han crossbred lambs (body weight = 32.5 ± 2.5 kg, 4–4.5 months old) were randomly allocated into two dietary treatments (*n* = 10 per group): a control group fed a basal diet (roughage to concentrate ratio of 45:55) or an AMO group supplemented with 56 mg/d of AMO per lamb. The trial consisted of a 15-day adaptation period followed by a 60-day experimental period. At the end of the trial, six lambs were randomly selected from each group for slaughter. Samples of the *longissimus thoracis* (LT) muscle were collected to determine meat quality traits, proximate composition, and amino acid profiles. Supplementation increased average daily gain by 6.6% and improved feed conversion ratio by 4.6% (*p* < 0.05), whereas feed intake and final body weight were not affected (*p* > 0.05). In LT, GR tissue depth and loin muscle area were increased (*p* < 0.05). Drip loss was reduced (*p* < 0.05), whereas proximate composition, cooking loss, and shear force remained unchanged (*p* > 0.05). The hydrolyzed amino acid composition and protein nutritional value of LT were not affected (*p* > 0.05). However, total free amino acid (FAA), total essential FAA, and the concentrations of free leucine, isoleucine, lysine, valine, phenylalanine, tyrosine, alanine, glutamic acid, glycine, and cysteine were increased by supplementation (*p* < 0.05). Moreover, flavor-related FAA, including umami-, sweet-, and bitter/sweet/sulfurous-related FAA were also increased by supplementation (*p* < 0.05). These results indicate that AMO improves growth efficiency and enhances LT meat quality, particularly by increasing flavor-related FAA, without altering protein nutritional characteristics.

## Simple Summary

Improving growth efficiency and meat quality in lamb production is important for sustainable animal farming. Antibiotics are commonly used in lamb production to improve animal health and growth performance, but concerns about residues and antimicrobial resistance have increased interest in natural alternatives. Plant-based feed additives are considered promising alternatives, although their effects on specific muscles and meat characteristics remain unclear. This study investigated the effects of adding essential oil extracted from *Allium mongolicum* Regel to the diet of finishing lambs, with a focus on the *longissimus thoracis* muscle, a major edible muscle closely associated with meat quality. The results showed that lambs receiving this supplement grew faster and used feed more efficiently, without changes in feed intake. In addition, muscle development in the *longissimus thoracis* was improved, and drip loss was reduced, indicating better meat quality. Although the basic protein composition of the muscle did not change, the levels of small compounds related to taste were increased, suggesting enhanced flavor potential. These findings indicate that this natural plant extract could improve both production efficiency and the quality of a key meat cut in lamb.

## 1. Introduction

In modern lamb production systems, improving meat quality, particularly meat flavor, while maintaining production efficiency has become a key research focus. Compared with traditional grazing systems, intensive feeding conditions, characterized by reduced physical activity and high-concentrate diets, often lead to alterations in meat quality [1,2]. In parallel, antibiotic feed additives have historically been used in livestock production to improve feed efficiency, reduce the incidence of subclinical diseases, and stabilize animal performance under intensive production conditions [3,4]. However, the long-term use of antibiotics has raised increasing concerns regarding drug residues in animal products and the emergence of antimicrobial resistance, which may threaten both animal and public health. Consequently, the use of antibiotic growth promoters has been restricted or banned in many countries [3,4]. Although acceptable productive performance can still be achieved in antibiotic-free systems through optimized management and nutrition strategies, natural feed additives with multiple bioactive functions are receiving increasing attention as sustainable alternatives. Under these circumstances, plant-derived bioactive compounds, owing to their natural origin and diverse physiological functions, have emerged as promising alternatives to antibiotics for improving growth efficiency and meat quality [5,6,7].

Meat flavor is the result of complex interactions among multiple components, among which amino acids not only determine protein nutritional properties but also serve as important precursors for flavor formation [8,9,10]. Based on their forms in muscle, amino acids can be classified into protein-bound (hydrolyzed amino acids) and free amino acids (FAAs). The former are mainly derived from structural proteins and generally remain relatively stable, showing limited responsiveness to short-term nutritional interventions [11,12,13]. In contrast, FAAs play a more direct role in flavor perception. Different FAAs contribute to distinct taste attributes [8,10,14]. Moreover, during thermal processing, FAAs participate in Maillard reactions and Strecker degradation, leading to the formation of various volatile compounds, including ketones, aldehydes, and sulfur-containing substances, thereby contributing to the characteristic flavor profile of lamb meat [15,16,17]. In addition to amino acid-derived compounds, lipid oxidation products and fatty acid composition also contribute substantially to meat aroma and flavor development. Moreover, interactions between lipid-derived volatiles and Maillard reaction products jointly shape the final sensory characteristics of cooked meat [17]. Compared with the relatively stable composition of structural proteins, FAA profiles are considered more responsive to nutritional regulation and, therefore, may represent an important target for improving lamb meat flavor quality.

Plant essential oils, as typical plant secondary metabolites, possess multiple biological activities, including antioxidant capacities, anti-inflammatory effects, and broad-spectrum antimicrobial efficacy [18,19,20,21]. In ruminant animals, previous work has indicated that essential oils can regulate rumen fermentation patterns and alter microbial community structure, thereby improving nutrient digestibility and utilization [22,23,24]. Most previous studies on essential oils in livestock production have mainly focused on animal health, antioxidant status, rumen fermentation, and productive performance, whereas comparatively less attention has been given to their effects on meat flavor-related components. Importantly, the metabolic effects of essential oils are not limited to energy metabolism but may also influence nitrogen metabolism. Previous studies have suggested that plant-derived active compounds can reduce excessive amino acid deamination, improve the efficiency of microbial protein synthesis, and enhance amino acid utilization and partitioning within the host [25,26]. In addition, essential oils may alter lipid metabolism and fatty acid deposition, including changes in unsaturated fatty acid profiles, which could further affect meat flavor characteristics [7,19]. Through these combined effects on rumen fermentation, oxidative status, protein turnover, and nutrient metabolism, plant-derived additives may ultimately influence the accumulation of flavor-related compounds in muscle tissue, including FAAs [27,28]. Consistent with this possibility, several studies in poultry and ruminants have reported that plant-based additives can increase the concentrations of certain flavor-related FAAs in muscle, thereby contributing to improved sensory quality of meat [7,12,29].

*Allium mongolicum* Regel, a perennial *Allium* species widely distributed in arid grassland regions, is rich in bioactive compounds such as polysaccharides, flavonoids, and volatile essential oils [30,31]. Previous studies have demonstrated that supplementation with *Allium mongolicum* Regel or its extracts can improve growth performance, antioxidant capacity, and meat quality in lambs, goats, and beef cattle to varying extents [7,32,33,34,35,36]. In terms of flavor regulation, existing research has primarily focused on its ability to reduce the accumulation of branched-chain fatty acids, thereby alleviating the characteristic mutton odor [37,38,39]. However, compared with lipid-derived flavor compounds, information regarding taste-related components, particularly FAA profiles in the *longissimus thoracis* (LT) muscle, remains limited. In addition, our previous study demonstrated that dietary supplementation with *Allium mongolicum* Regel essential oil (AMO) at a comparable dosage (approximately 56 mg/d per lamb) improved rumen fermentation characteristics and feed digestibility in finishing lambs [22], suggesting a potential metabolic link between rumen regulation and muscle nutrient deposition.

Therefore, we hypothesized that dietary supplementation with AMO could improve meat quality and alter flavor-related FAA profiles in the LT through the modulation of rumen fermentation and nutrient metabolism. Accordingly, the present study was conducted to evaluate the effects of dietary AMO supplementation on growth performance, meat quality, and amino acid profiles in the LT of finishing lambs.

## 2. Materials and Methods

### 2.1. Essential Oil Extraction

The dried leaves of *Allium mongolicum* Regel were obtained from a cultivation base (Haohai Bio Co., Ltd., Alxa League, China). The essential oil was prepared by hydrodistillation using a Clevenger-type apparatus (RV8, IKA, Guangzhou, China) [22]. Briefly, dried leaf powder was mixed with distilled water at a ratio of 1:5 (*w*/*v*) and distilled for 3 h. The collected oil fraction was dehydrated with anhydrous sodium sulfate and stored at 4 °C until use. According to previous GC–MS analysis, the major components included anethole (37.6%), aromatic hydrocarbons (30.5%), and gingerol (12.6%) [22].

### 2.2. Animals, Housing, and Experimental Design

Twenty Dorper × Han crossbred lambs, aged 4 to 4.5 months, with similar initial body weight (32.5 ± 2.5 kg), were enrolled in the feeding trial and randomly allocated to two treatments, with ten animals per group. The lambs were housed in individual pens. Each pen was situated within a semi-open shed for natural ventilation and shade. Each pen was equipped with stainless-steel feeders and automatic waterers to provide free access to feed and water throughout the experiment. The two dietary treatments consisted of a basal diet alone (control) or the same basal diet supplemented with 56 mg AMO per lamb per day. The inclusion level of AMO was selected according to our previous in vivo feeding trial [22], in which this dosage improved rumen fermentation characteristics and nutrient digestibility in lambs. The basal diet was formulated to meet the nutrient requirements of growing-finishing sheep for maintenance and growth according to NRC, (2007) [40], and the ingredient composition and nutrient contents are shown in Table 1. Prior to feeding, AMO was first pre-blended with the concentrate portion and subsequently mixed thoroughly into the total mixed ration. Lambs were offered feed twice daily at 07:00 and 18:00, in approximately equal amounts. The quantity of feed provided was adjusted daily to maintain orts at approximately 10% of the amount offered, thereby ensuring ad libitum intake. Both feed offered and orts were recorded for the calculation of feed intake. The entire trial lasted 75 days, comprising a 15-day adaptation phase and a subsequent 60-day experimental period during which performance data and samples were collected.

### 2.3. Growth Performance

At the end of the 15-day adaptation period, body weight measured on day 1 of the experimental period was considered the initial body weight. Individual body weight was measured before the morning feeding (07:00) using a digital platform scale (PS200; Delixi Electric Co., Ltd., Jinhua, China). Body weight was subsequently recorded at 15-day intervals, and average daily gain (ADG) was calculated for each interval. Changes in body weight and ADG over experimental periods were used for subsequent analysis and graphical presentation. Daily dry matter intake (DMI) was calculated based on recorded feed intake and the dry matter content of the total mixed ration. The dry matter content of the feed and orts was determined according to AOAC method (934.01) [41]. Average daily DMI was determined for each 15-day interval. Feed conversion ratio (FCR) was calculated as the ratio of DMI to ADG. Temporal changes in DMI and FCR were also analyzed and presented.

### 2.4. Slaughter Procedures and Carcass Evaluation

At the end of the 60-day trial, Six lambs from each treatment group were randomly selected for carcass and meat quality evaluation. Selected animals were fasted overnight with free access to water prior to slaughter. Slaughter procedures were conducted at a commercial abattoir following standard commercial practices. Briefly, lambs were electrically stunned, exsanguinated by severing the jugular vein and carotid artery, skinned, and eviscerated. Hot carcass weight was recorded immediately after slaughter, and dressing percentage was calculated as the ratio of hot carcass weight to final live body weight. Carcass tissue depth measured 11 cm from the midline over the 12th rib (GR value), backfat thickness, and loin muscle area were determined according to previously described methods [42,43]. Briefly, backfat thickness was measured at the interface between the 12th and 13th ribs using a vernier caliper, whereas loin muscle area was determined by tracing the exposed muscle surface after transverse sectioning at the 12th rib.

### 2.5. Meat Quality Analysis of LT Muscle

#### 2.5.1. Sampling

LT muscle samples were excised from the left side of each carcass at the region spanning the 7th to 12th ribs at approximately 45 min postmortem. Each LT sample was subsequently separated into three subsamples for different analyses. One subsample was snap-frozen in liquid nitrogen, shipped to the lab, and stored at −80 °C for subsequent determination of hydrolyzed and free amino acid contents. A second subsample was shipped to the lab on dry ice and preserved at −20 °C for proximate composition analysis. The remaining subsample was kept at 4 °C for on-site evaluation of physical meat quality traits.

#### 2.5.2. Physical Properties and Proximate Analysis

Drip loss was determined by suspending LT muscle samples in sealed containers at 4 °C for 24 h, and the percentage weight loss was calculated. Cooking loss was measured after samples were heated in a water bath until the internal temperature reached 75 °C, followed by cooling to room temperature. Shear force was evaluated using a Warner–Bratzler shear device (RH-N50, Runhu Instrument Co., Ltd., Guangzhou, China) after cooking. Detailed analytical procedures followed previously published protocols [44].

The moisture content of the LT muscle samples was quantified by assessing the reduction in sample mass following the lyophilization process. Proximate composition, including crude protein (AOAC 928.08), ether extract (AOAC 960.39), and ash content (AOAC 942.05), were analyzed according to AOAC methods [45,46,47].

#### 2.5.3. Amino Acid Composition

Hydrolyzed amino acids were determined according to AOAC method 994.12 [48]. Approximately 50 mg of lyophilized LT muscle was placed into a hydrolysis tube and mixed with 10 mL of 6 mol/L HCl. The tube was then purged with nitrogen, tightly sealed with a Teflon-lined cap, and subjected to acid hydrolysis at 110 °C for 22–24 h. After the hydrolysis process, the mixture was cooled to room temperature and subsequently filtered through quantitative filter paper. The filtrate was brought to a final volume of 50 mL with distilled water. A 1.0 mL aliquot was collected, evaporated to dryness under vacuum, redissolved in 2.5 mL of 0.02 mol/L HCl, and passed through a 0.22 μm membrane filter before instrumental analysis.

FAAs were extracted according to Yu et al. [49]. In brief, approximately 0.2 g of lyophilized LT muscle tissue was homogenized in 1.5 mL of 0.02 mol/L HCl and vortexed for 15 min. The homogenate was centrifuged at 3000× *g* for 15 min at a temperature of 4 °C. Following this, a 0.5 mL aliquot of the supernatant was collected and treated with 10% sulfosalicylic acid. After overnight incubation at 4 °C, the suspension was centrifuged again at 14,000× *g* for 20 min at a temperature of 4 °C. The supernatant was then collected and processed through a 0.22 μm membrane filter for chromatographic analysis.

Amino acid concentrations were determined using an automated amino acid analyzer (L-8900, Hitachi, Tokyo, Japan). Separation was performed using a sodium-form cation-exchange column (4.6 mm × 60 mm) with a standardized injection volume of 20 μL. For the chromatographic elution, the mobile phase consisted of a series of citrate buffers with different pH values, delivered at a constant flow rate of 0.40 mL/min while the column environment was maintained at 57 °C. Post-column derivatization was performed using ninhydrin solution at a delivery rate of 0.35 mL/min with the reaction coil maintained at 135 °C. Amino acids were identified and quantified using a mixed external standard (013-08391, Wako Pure Chemical Industries, Ltd., Osaka, Japan) with dual-wavelength detection at 420 and 570 nm. Absorbance at 420 nm was used for the determination of proline, whereas detection at 570 nm was applied to all other amino acids.

### 2.6. Statistical Analysis

The experiment was conducted using a completely randomized design, with the individual lamb as the experimental unit. Growth performance data were analyzed using all animals (*n* = 10 per treatment), whereas carcass traits, meat quality parameters, proximate composition, and amino acid profiles were analyzed using data from six randomly selected lambs per treatment (*n* = 6 per treatment).

Statistical analysis of the collected data was performed using SAS Studio On Demand for Academics version (SAS Institute Inc., Cary, NC, USA) via the MIXED procedure. The data were analyzed according to the following model: Yij = µ + Ti + eij, where Yij is the dependent variable, µ is the overall mean, Ti is the fixed effect of dietary treatment and eij is the residual error. Prior to analysis, residual normality and homogeneity of variance were evaluated using the Shapiro–Wilk test, Q–Q plots, and Levene’s test, respectively. No violations of model assumptions were detected (*p* > 0.05), and therefore no data transformation was required. Results are reported as least squares means accompanied by the standard error of the mean (SEM). Additionally, visual representations of growth dynamics and taste-active FAA concentrations were constructed utilizing Origin 2026 software (OriginLab, Northampton, MA, USA), with data points in the illustrations depicted as mean values and error bars representing the standard error.

## 3. Results

### 3.1. Growth Performance and Carcass Characteristics

**Figure 1:**
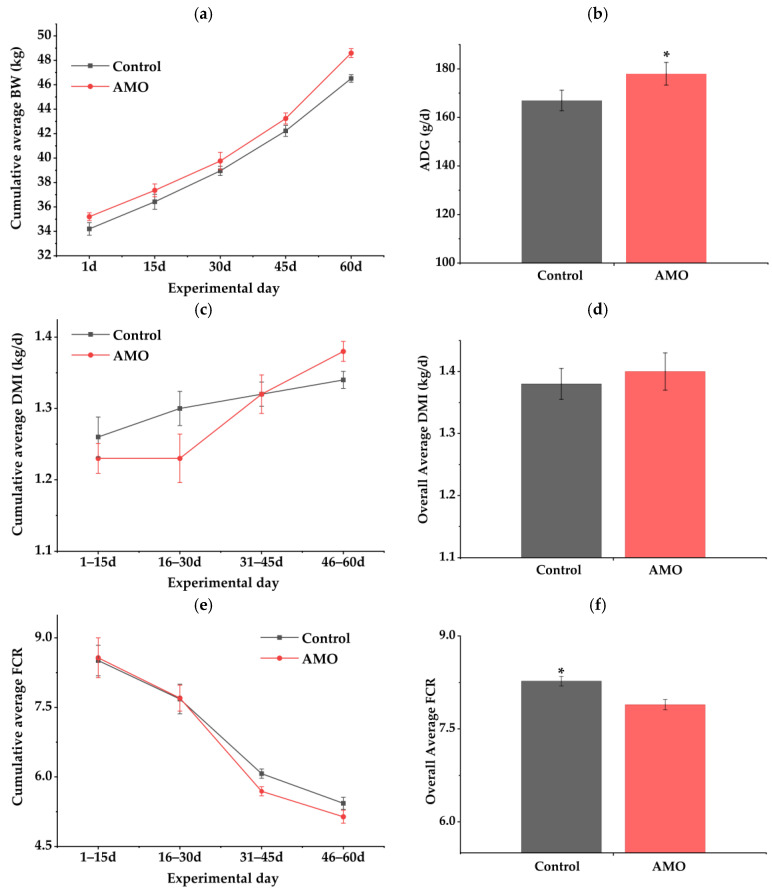
Growth performance parameters of finishing lambs receiving diets with or without *Allium mongolicum* Regel essential oil. (**a**) cumulative average body weight (BW) over the 60-day experimental period; (**b**) average daily gain (ADG); (**c**) cumulative average daily dry matter intake (DMI) over the 60-day experimental period; (**d**) overall average daily DMI; (**e**) cumulative average feed conversion ratio (FCR) over the 60-day experimental period; (**f**) overall average FCR. Control, a basal diet; AMO, basal diet supplemented with 56 mg *Allium mongolicum* Regel essential oil per day each animal. Values are presented as means ± standard error. Bars marked with * indicate significant differences between treatments (*p* < 0.05).

Throughout the experimental period, AMO supplementation increased daily weight gain. As shown in Figure 1, dry matter intake was lower in the first 45 days but higher during the final phase of the trial, corresponding to an improvement in feed efficiency during the later stage of the trial. Overall (Table 2), dietary AMO supplementation increased ADG by 6.6% (*p* = 0.04) compared with the control group. No significant difference was detected in total dry matter intake (*p* = 0.36), and overall FCR decreased by 4.6% (*p* = 0.01).

Regarding carcass traits, AMO increased GR value (*p* = 0.01) and loin muscle area (*p* = 0.04) compared with the control group, indicating enhanced carcass lean traits. No significant differences were observed between the two groups regarding final body weight, carcass weight, dressing percentage, or backfat thickness (*p* ≥ 0.14; Table 2).

### 3.2. Proximate Compositions and Physical Characteristics of the LT Muscle

Dietary AMO supplementation had no effect on the moisture, crude protein, ether extract, or ash contents of the LT muscle (*p* ≥ 0.17; Table 3). However, lambs receiving AMO had significantly lower drip loss (*p* = 0.01), whereas cooking loss and shear force were not affected by treatments (*p* ≥ 0.14; Table 3).

### 3.3. Hydrolyzed Amino Acid Content and Essential Amino Acid Scores of the LT Muscle

Compared with the control group, the dietary inclusion of AMO did not significantly alter the concentrations of total hydrolyzed amino acids, essential amino acids, non-essential amino acids, or any individual amino acid in the LT muscle (*p* ≥ 0.07; Table 4).

When assessed against the FAO/WHO/UNU (2007) reference pattern for adults [50,51], the essential amino acid score and essential amino acid index did not differ significantly between the two groups (*p* ≥ 0.08; Table 5). Both the AMO and control groups had scores exceeding 100, confirming that both dietary treatments met the essential amino acid requirements of adults. In both groups, valine had the lowest score, followed by leucine and isoleucine, indicating that branched-chain amino acids (BCAAs) are the limiting amino acids in lamb meat (Table 5).

### 3.4. FAA Content and Taste-Related FAA Content of the LT Muscle

Dietary AMO supplementation significantly influenced the free amino acid profile of the LT muscle (Table 6). Specifically, regarding essential amino acids, the AMO group exhibited significantly higher concentrations of leucine, isoleucine, lysine, valine, and phenylalanine (*p* ≤ 0.047). For non-essential amino acids, significantly higher concentrations of alanine, glutamic acid, glycine, cysteine, and tyrosine were observed in the AMO group (*p* ≤ 0.043). Overall, total FAA, total free essential amino acids, and total free BCAA were significantly increased in the LT muscle of AMO-supplemented lambs (*p* ≤ 0.048).

**Figure 2:**
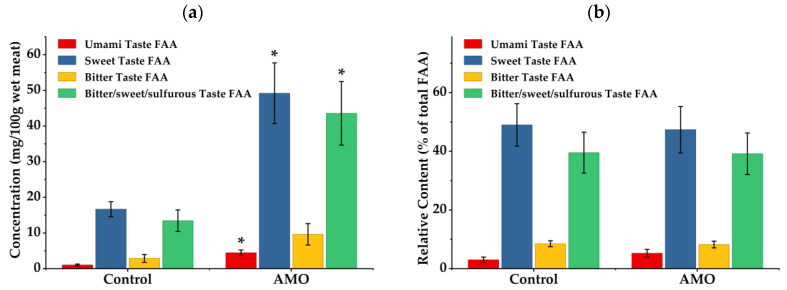
Taste-related free amino acid (FAA) contents of *longissimus thoracis* muscles in sheep affected by *Allium mongolicum* Regel essential oil supplementation in diet (*n* = 6). (**a**) Concentration of taste-related FAA, (**b**) Relative Content of taste-related FAA. Umami taste FAA = glutamic acid + aspartic acid, sweet taste FAA = threonine + glycine + alanine, Bitter taste FAA = isoleucine + leucine + tyrosine + phenylamine + histamine, Bitter/sweet/sulfurous taste FAA = cysteine + valine + methionine + lysine + arginine + proline [8]. Control, a basal diet; AMO, basal diet supplemented with *56* mg *Allium mongolicum* Regel essential oil per day each animal. Graph bars with * indicate groups with significantly higher values (*p* < 0.05).

The concentrations of taste-related FAAs within the LT muscle, including sweet-, umami-, and bitter/sweet/sulfurous-related amino acids, were significantly increased in the AMO supplemented group compared with the control group (*p* = 0.027, 0.014, and 0.044; Figure 2a). However, when expressed as a percentage of total FAA, no significant difference in the distribution of taste-related FAAs was observed between the two treatments (*p* ≥ 0.14; Figure 2b).

## 4. Discussion

Non-antibiotic feed additives, including plant essential oils and other plant-derived bioactive compounds, have often been reported to have no significant effects on feed intake or final body weight but can improve ADG and FCR [12,19,52]. A similar pattern was observed in the present study, suggesting that the improvement in growth performance may be associated with enhanced feed utilization efficiency rather than increased feed intake. Previous studies have shown that supplementation with different forms of *Allium mongolicum* Regel, including dried powder, water-soluble extracts, and flavonoid preparations, can improve ADG and FCR in finishing lambs to varying extents [34,35,37], which is consistent with the findings of the present study. Although ADG was significantly improved by AMO supplementation, no significant difference was observed in final body weight. This discrepancy may be related to the relatively short experimental period and the inherent variation in body weight among individual animals. Similar patterns have been reported in studies evaluating plant-derived feed additives in ruminants, where improvements in growth efficiency did not always translate into significant differences in final body weight [12,52].

Previous studies have suggested that *Allium*-derived additives and other plant essential oils may improve growth performance by modulating rumen fermentation patterns, particularly enhancing propionate production and improving antioxidant and immune status [7,19,53,54]. These changes may contribute to improved nutrient utilization and muscle deposition. However, inconsistent effects on ADG and FCR have also been reported for different forms of *Allium mongolicum* extracts [33,55], which may be related to differences in extract composition, bioactive constituents, and supplementation levels. In the present study, the AMO preparation contained a relatively high proportion of volatile compounds, which may be associated with its positive effects on growth efficiency. However, the underlying mechanisms require further investigation. In addition, further studies evaluating multiple supplementation levels are warranted to better characterize dose-dependent responses of AMO in finishing lambs.

Although AMO did not significantly affect final body weight, it increased GR value and loin muscle area, indicating potential improvements in lean tissue deposition and carcass composition. Similar effects have been reported for *Allium mongolicum* extracts and other plant-derived additives in finishing lambs [12,35,37]. One possible explanation is that plant-derived bioactive compounds alter rumen fermentation patterns, thereby improving energy utilization and supporting muscle deposition [19,22,53]. These findings suggest that AMO may be associated with nutrient partitioning toward lean tissue accretion rather than fat deposition.

In addition, AMO supplementation significantly reduced drip loss in the LT muscle, suggesting a potential improvement in meat water-holding capacity. Drip loss is closely associated with muscle cell membrane integrity and oxidative stability. Previous studies have demonstrated that *Allium mongolicum* and its extracts possess considerable antioxidant activity and can improve systemic antioxidant status in finishing lambs [31,56]. Therefore, the reduced drip loss observed in the present study may be partly associated with improved oxidative status and reduced muscle cell damage. The underlying mechanisms require further investigation.

Dietary supplementation with AMO did not significantly affect the hydrolyzed amino acid composition of the LT muscle, which is consistent with previous studies showing that protein-bound amino acid profiles in muscle are relatively stable under different nutritional interventions [7,11,13]. Similarly, essential amino acid scores and essential amino acid index remained unchanged between treatments, indicating that AMO did not alter the overall nutritional quality of muscle protein. These findings suggest that the effects of AMO were not associated with changes in structural protein composition but may be related to alterations in metabolically active amino acid pools, such as FAAs.

In contrast to the hydrolyzed amino acid profile, AMO supplementation significantly increased the concentrations of total FAA and total essential FAA in the LT muscle. FAAs are metabolically active compounds involved in protein turnover and muscle metabolism [57,58,59]. Previous studies have suggested that plant-derived bioactive compounds may increase amino acid availability by modulating rumen fermentation, reducing excessive amino acid deamination, and enhancing microbial protein synthesis [27,28]. Similar increases in muscle FAA concentrations have also been reported following supplementation with other plant-derived additives, including *Cannabis sativa* residues and thyme essential oil [12,29,60]. Therefore, the elevated FAA levels observed in the present study may be associated with improved amino acid utilization and metabolic regulation.

From the perspective of meat quality, FAAs are key precursors determining flavor characteristics, and their variation has a direct impact on sensory attributes. Different FAA contribute to distinct taste modalities: glutamic acid and aspartic acid are primarily associated with umami taste, glycine and alanine contribute to sweetness, whereas BCAAs, such as valine, leucine, and isoleucine, are closely related to bitterness and subsequent flavor formation [8,10,14]. In the current study, AMO significantly enhanced the concentrations of several flavor-related amino acids, including alanine, glutamic acid, glycine, and BCAA. Correspondingly, the total levels of umami, sweet, and bitter/sweet/sulfurous taste FAAs were also markedly elevated, suggesting that AMO enhances the reservoir of flavor precursors in lamb meat. Furthermore, during thermal processing, FAAs can undergo Maillard reactions and Strecker degradation, leading to the formation of various volatile compounds. For example, sulfur-containing compounds derived from methionine and aldehydes generated from BCAAs are known to play important roles in the characteristic flavor of lamb [15,16,17,61]. Although the relative proportions of different taste-related amino acids within total FAA were not significantly altered in this study, the increase in their absolute concentrations may still contribute to enhanced flavor intensity and overall sensory quality. Collectively, these findings suggest that AMO may improve the flavor potential of lamb meat by selectively elevating FAA levels, without altering the fundamental nutritional value of muscle protein, which may represent one of the mechanisms underlying its beneficial effects on meat quality.

## 5. Conclusions

In conclusion, dietary supplementation with 56 mg/d of *Allium mongolicum* Regel essential oil per lamb improved growth performance in finishing lambs as reflected by increased average daily gain and improved feed conversion efficiency. Furthermore, the supplementation improved carcass traits and meat quality by increasing GR value and loin muscle area while reducing drip loss. Although no changes were observed in hydrolyzed amino acid composition or protein nutritional value, supplementation significantly increased the concentrations of flavor-related free amino acids, thereby potentially enhancing the flavor profile of lamb meat.

## Figures and Tables

**Table 1:** Composition and nutrient levels of the experimental diet (dry matter basis).

Items	Numerical Value
Ingredients, %	
Alfalfa	27.78
Whole corn silage	15.52
Gourd seed skin	12.15
Corn	19.25
Wheat bran	4.29
Distillers dried grains and soluble	4.33
Sunflower seed meal	6.09
Flaxseed meal	4.62
Red dates	1.68
Limestone	1.48
Dicalcium phosphate	0.69
Sodium chloride	0.72
Mineral and vitamin premix ^1^	1.40
Nutrient levels ^2^	
Digestible energy, MJ/kg	16.83
Crude protein, %	15.32
Ether extract, %	3.09
Neutral detergent fiber, %	50.98
Acid detergent fiber, %	29.69
Calcium, %	1.02
Phosphorous, %	0.55

^1^ The mineral and vitamin premix supplied the following per kg of diet: 25 mg Fe, 29 mg Zn, 8 mg Cu, 30 mg Mg, 0.04 mg I, 0.1 mg Co, 3200 IU vitamin A, 1200 IU vitamin D, and 20 IU vitamin E. ^2^ Digestible energy was derived by calculation, whereas all other values were determined analytically.

**Table 2:** Effects of *Allium mongolicum* Regel essential oil supplementation in diet on growth performance and carcass traits in sheep.

Variables	Treatments ^1^		SEM ^2^	*p*-Value
	Control	AMO		
Overall growth performance 1–60 d				
Initial body weight, kg	34.2	35.2	0.73	0.38
Average dry matter intake, kg/d	1.38	1.40	0.03	0.36
Average daily gain, g/d	167	178	4.25	0.04
FCR ^3^	8.27	7.89	0.08	0.01
Final body weight, kg	46.6	48.6	3.10	0.54
Carcass traits				
Carcass weight, kg	20.9	22.3	1.28	0.29
Dressing percentage, %	44.9	45.7	0.93	0.27
GR value, mm	4.30	6.60	0.69	0.01
Backfat thickness, mm	4.43	5.07	0.57	0.12
Loin muscle area, cm^2^	17.4	25.8	0.56	0.01

^1^ Treatments: Control, a basal diet; AMO, basal diet supplemented with 56 mg *Allium mongolicum* Regel essential oil per day each animal. ^2^ SEM means standard error of the mean. ^3^ FCR means feed conversion ratio = Average dry matter intake/Average daily gain.

**Table 3:** Effects of *Allium mongolicum* Regel essential oil supplementation in diet on proximate compositions and physical characteristics of *longissimus thoracis* in sheep.

Variables	Treatments ^1^		SEM	*p*-Value
	Control	AMO		
Proximate compositions (g/kg muscle, based on wet weight)				
Moisture	718.71	718.91	3.49	0.98
Crude protein	188.32	193.67	2.77	0.17
Ether extract	51.79	44.90	3.45	0.38
Ash	37.69	41.88	2.08	0.37
Physical characteristics				
Drip loss (%)	3.95	3.40	0.28	0.01
Cooking loss (%)	39.23	38.44	1.06	0.49
Shear force (N)	30.84	35.18	2.78	0.14

^1^ Treatments: Control, a basal diet; AMO, basal diet supplemented with 56 mg *Allium mongolicum* Regel essential oil per day each animal.

**Table 4:** Effects of *Allium mongolicum* Regel essential oil supplementation in diet on total amino acids content following hydrolysis of *longissimus thoracis* in sheep (mg/g wet muscle).

Variables	Treatments ^1^		SEM	*p*-Value
	Control	AMO		
Essential amino acids, EAA				
Arginine	12.31	11.73	0.49	0.42
Histidine	6.60	5.81	0.27	0.07
Leucine	15.80	14.81	0.63	0.27
Isoleucine	8.50	7.90	0.38	0.29
Lysine	16.83	15.68	0.71	0.27
Methionine	5.63	5.20	0.20	0.16
Phenylalanine	8.63	8.47	0.48	0.82
Threonine	9.02	5.58	0.34	0.38
Valine	8.78	8.47	0.38	0.58
Total EAA	92.10	86.65	3.67	0.32
Non-essential amino acids, NEAA				
Alanine	11.22	10.65	0.41	0.34
Aspartic acid	16.74	15.18	0.72	0.16
Glutamic acid	28.71	27.03	1.02	0.27
Glycine	7.81	7.09	0.35	0.17
Serine	7.37	6.87	0.25	0.19
Tyrosine	5.76	5.25	0.26	0.20
Proline	9.89	10.05	0.56	0.85
Cysteine	1.40	1.45	0.14	0.83
Total NEAA	88.89	83.55	2.99	0.24
Total amino acid	180.99	168.19	6.59	0.27
SAA ^2^	7.03	6.65	0.29	0.37
BCAA ^3^	33.09	31.19	1.36	0.35

^1^ Treatments: Control, a basal diet; AMO, basal diet supplemented with 56 mg *Allium mongolicum* Regel essential oil per day each animal. ^2^ SAA (sulfur-containing amino acids) = Methionine + Cysteine. ^3^ BCAA (branched-chain amino acid) = Leucine + Isoleucine + Valine.

**Table 5:** Effects of *Allium mongolicum* Regel essential oil supplementation in diet on essential amino acid score and essential amino acid index of *longissimus thoracis* protein in sheep (*n* = 6).

Variables	Reference Protein ^1^	Treatments ^2,3^		SEM	*p*-Value
		Control	AMO		
Histidine	15 mg/g	244	227	6.13	0.08
Leucine	59 mg/g	148	148	0.72	0.74
Isoleucine	30 mg/g	156	155	2.89	0.76
Lysine	45 mg/g	206	205	3.48	0.80
Methionine + Cysteine	22 mg/g	177	177	3.40	0.89
Phenylalanine + Tyrosine	38 mg/g	209	212	3.38	0.59
Threonine	23 mg/g	217	219	2.43	0.53
Valine	39 mg/g	124	128	2.00	0.21
EAAI ^4^	100	181	180	0.98	0.65

^1^ The reference protein for average adult is adapted from FAO/WHO/UNU 2007 [50,51]. ^2^ Values for individual amino acids under ‘Control’ and ‘AMO’ are scores calculated relative to the Reference protein and are dimensionless. ^3^ Treatments: Control, a basal diet; AMO, basal diet supplemented with 56 mg *Allium mongolicum* Regel essential oil per day each animal. ^4^ EAAI means essential amino acid index.

**Table 6:** Effects of *Allium mongolicum* Regel essential oil supplementation in diet on free amino acids content of *longissimus thoracis* in sheep (mg/100 g wet muscle; *n* = 6).

Variables	Treatments ^1^		SEM	*p*-Value
	Control	AMO		
Essential amino acids, EAA				
Arginine	1.732	3.933	0.712	0.061
Histidine	0.421	1.374	0.641	0.320
Leucine	0.763	2.561	0.504	0.034
Isoleucine	0.442	1.521	0.303	0.034
Lysine	7.614	32.062	7.212	0.043
Methionine	0.661	0.970	0.291	0.470
Phenylalanine	0.039	3.092	0.582	0.033
Threonine	3.351	12.363	2.594	0.039
Valine	0.842	2.854	0.491	0.019
Total EAA	15.865	60.730	11.890	0.047
Non-essential amino acids, NEAA				
Alanine	9.181	27.492	4.922	0.030
Aspartic acid	0.054	0.062	0.025	0.821
Glutamic acid	0.972	4.381	0.761	0.013
Glycine	2.832	7.834	1.262	0.023
Serine	1.294	1.503	0.392	0.720
Tyrosine	0.306	1.082	0.391	0.190
Proline	2.312	3.124	0.732	0.460
Cysteine	0.283	0.654	0.111	0.043
Total NEAA	16.773	60.702	11.973	0.051
Total amino acid	33.099	106.862	20.453	0.048
SAA ^2^	0.944	1.624	0.30	0.160
BCAA ^3^	2.047	6.936	1.27	0.026

^1^ Treatments: Control, a basal diet; AMO, basal diet supplemented with 56 mg *Allium mongolicum* Regel essential oil per day each animal. ^2^ SAA (sulfur-containing amino acids) = Methionine + Cysteine. ^3^ BCAA (branched-chain amino acid) = Leucine + Isoleucine + Valine.

## Data Availability

The original contributions presented in this study are included in the article. Further inquiries can be directed to the corresponding authors.

## Abbreviations

The following abbreviations are used in this manuscript:

AMO	*Allium mongolicum* Regel essential oil
LT	*longissimus thoracis*
FAA	free amino acid
ADG	average daily gain
DMI	dry matter intake
FCR	feed conversion ratio
BCAA	branched-chain amino acid
